# Students' academic burnout in Iranian agricultural higher education system: the mediating role of achievement motivation

**DOI:** 10.1016/j.heliyon.2020.e04960

**Published:** 2020-09-21

**Authors:** Mahnaz Tajeri Moghadam, Enayat Abbasi, Zahra Khoshnodifar

**Affiliations:** a Department of Agricultural Extension and Education, Tarbiat Modares University, Tehran, Iran; b Education & Agricultural Extension Department, Higher Educational Complex of Saravan, Saravan, Iran

**Keywords:** Education, Psychology, Academic burnout, Achievement motivation, Proper workload, Agricultural students, Higher education, Iran

## Abstract

Agricultural higher education is one of the important context in which students may be face with educational burnout during their studies due to their conditions, such as the nature of the field of study, lack of graduates' employment, and reduction in motivation. This research aimed to investigate the factors underpinning the student's academic burnout of Iranian agricultural higher education system. The research was a kind of descriptive-correlational that has done through a survey. The statistical population composed of all students at all educational levels in agricultural faculties of the Iranian state-run universities (N = 236,973). Accordingly, 386 people were selected using the Krejcie and Morgan's tables and stratified random sampling method with proportional to size. A researcher-made questionnaire with 104 questions arranged in six parts used for data collection. The validity and reliability of the questionnaire were confirmed by calculating the Cronbach's alpha, average variance extracted (AVE) and composite reliability (CR). According to the results, the variables of high workload and achievement motivation were the most influential factors on academic burnout, respectively. This study's results can be a useful step for policymakers and planners in the agricultural higher education system to prevent student's academic burnout and remove obstacles to dynamic academic achievement.

## 1 Introduction

Enhancing quality of an educational system depends on quality of its components. Students play a key role in any educational system (as educational system inputs) to meet the goals of the system [1]. To be more thriving, an educational system needs to consider this community from an educational perspective. Most countries spend a significant amount of their national income on education, but the students entering the higher education system where they supposed to show a proper academic motivation and achievement [1] may not mostly have the required productivity and may suffer from academic burnout for many reasons. Burnout is a state of mental and emotional exhaustion contributed by the chronic stress syndrome such as role-bearing, pressure and time restraints, and lack of required resources for fulfilling tasks and duties [2].

Academic burnout signals the decline of an individual's aptitude to adjust himself/herself to the stressful factors of education [3]. In general, academic burnout includes three areas of academic fatigue, academic cynicism, and academic inefficacy. It makes students feel tired of doing homework and studying. It, also, results in pessimistic attitudes toward studying and educational contents and an unhealthy sense of education. Academic burnout leads to the loss of students' suitable academic performance and aggravates their concerns about committing mistakes in homework [4].

Research in different countries (South Korea, Philippines, Nigeria, Canada, etc.) on academic burnout has demonstrated that students in these countries are also suffering from this problem so that academic burnout clearly affects their grades as well as their relationship with the university [5]. Academic burnout also reduces students' motivation [6]. Thus, there is no sense of responsibility and accountability against their poor performance [7]. Moreover, such issues as the teaching and learning ambiance, high workload [8], social support and gender [9], the field of study [10] and quality of learning activities can affect students' academic burnout.

Academic burnout has plagued Iranian students as well [11]. The degree of academic burnout in students can be influenced by various factors such as quality of the university environment as well as academic and social cohesion [12], the quality of learning experiences [13], educational justice [14], and academic motivation [15], and it can affect academic performance, student commitment to educational affairs, interest in continuing education and academic participation after graduation [16].

Agricultural higher education is one of the important context in which students may be face with educational burnout during their studies due to their conditions, such as the nature of the field of study, lack of graduates' employment, and reduction in motivation [17]. For instance, the semester failure rate at the one of the Iranian agricultural higher education institute in 2012 was 4%, which increased by 15% in 2015. The number of dropouts was also 3% in 2012, but it increased by 5% in 2015 [18]. Agricultural faculties, as the subordinate of higher education in agriculture, are not efficient enough despite their largest share in use of the financial resources of the universities [19]. However, a declining trend has been recently observed in both the demand to start education at many agricultural colleges in Iran and in the demand for higher education in this field. In fact, it comes across that the interest in this field has waned after admission in Konkoor examination^1^ among applicants, and even many students stop studying in this field despite having passed some agricultural courses. According to available evidence, 247 students have refused to keep studying in various agricultural fields and disciplines at one of the Iranian universities during the academic years of 2011–2013 [20].

## 2 Literature review

In recent years, many studies have conducted about job burnout. Most studies on burnout have conducted on specific situations such as healthcare workers [21] and sales persons and nurses [22], came to called occupation burnout [23]. However, the burnout variable has extended to educational situations and learning environments [24].

In recent years, academic burnout in students for different reasons including understand different students' behavior such as academic achievement during education, affecting students' relationships with their college and university, and affecting students' enthusiasm for continuing education has turned into a significant research subject at universities [25]. Academic burnout has studied in different fields, but few studies have been done on agricultural education. The lack of research in agricultural faculties has made this research necessary and it can be strengthen the theoretical knowledge in the agricultural sector. In addition, university officials can benefit from the research results to make some changes in the resources, content, student-teacher relationship, educational system and university environment to prevent academic burnout in students, as well as many other academic problems of students such as academic dropout, request for changing field of study, multiple failure in semesters, ask for extra academic years, etc.

There are different factors affecting extend of students' academic burnout. For instance, Marzooghi, Heidari, & Heidari [14] concluded that improving educational justice (student-teacher relationship) decreases academic burnout in a variety of aspects (academic cynicism and inefficacy). Researchers investigated the relationship of hope for employment and academic motivation with academic burnout in their research on students' points of view in Medical Science University of Shahroud [26]. According to their results, there is a significant relationship between hope for employment and academic motivation with academic burnout.

A study on predicting academic burnout based on academic quality of life and hope for employment among university students showed a significant negative correlation between academic quality of life (QAL) and hope for employment in students with academic burnout [27]. Another group of researchers focused on the relationship between motivation and academic burnout among nursing and paramedical students, and found a negative significant relationship between academic burnout and achievement motivation so that the motivated students were more prone to burnout [11]. Academic burnout significantly decreased with the increase in achievement motivation [28]. Meriläinen [8] investigated factors affecting burnout among Finnish university students with a focus on learning environment, achievement motivation and the meaning of life and concluded that there was a significant relationship between quality of teaching and learning environment (teacher–student relationship, the quality of teaching, deep approach to learning, evaluation, pedagogical counseling, psychological contract, social relations between students, and usability of studies) and both factors of workload and achievement motivation. Also, based on the results of this study, there was a positive and negative relationship between high workload and achievement motivation and academic burnout dimensions, respectively. Researchers concluded that the quality of teaching and learning environment was the most effective factor of academic burnout and the achievement motivation, including success in studying, appreciation of university education, and ability beliefs was negatively associated with academic burnout [29]. Chang et al. [30] concluded that motivation variables had a significant moderate impact on the relationship between perfectionism and academic burnout symptoms. The variable of achievement motivation is also affected by variables such as students' effort, specialty and abilities [39].

Quality of teaching and learning environment is considered as one of the factors that students face. This component includes teacher–student relationship, the quality of teaching, deep approach to learning, evaluation, pedagogical counseling, psychological contract, social relations between students, and usability of studies [28, 29]. In general, quality of teaching and learning environment is a determining factor in motivating students to progress. The effective and positive quality of teaching and learning environment contributes to greater integration and academic progress. Lack of this environment makes it harder to meet these goals [32]. According to what has been said, it is necessary to consider students' achievement of knowledge and skills and to improve in their academic progress and performance. Meanwhile, in recent years Iran's higher education system has been faced with a quantitative and uncontrolled development of the number of students irregard to their capacities. Inequality and unfair distribution of resources and facilities, constant changes in educational and managerial policies, and high recruitment of students and faculty members regardless of their needs are other challenges of this system [33]. Lack of motivation, confusion and fatigue, uninterested and finally academic burnout among students are the results of these problems. Solving these challenges requires effective research. The lack of research in agricultural higher education has made this research necessary and can contribute to theoretical knowledge in the agricultural sector. Therefore, it is important and necessary to identify factors affecting students' academic burnout and provide solutions to reduce it.

### 2.1 Conceptual framework

**Figure 1:**
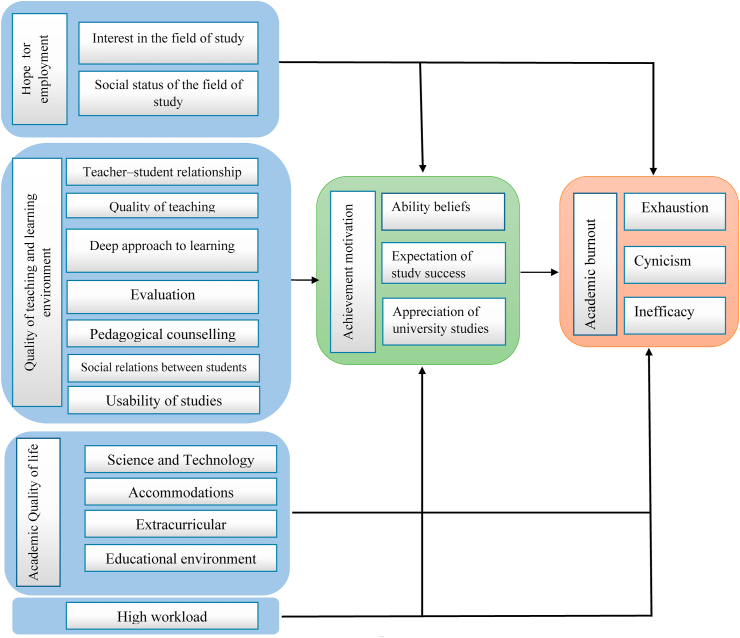
The conceptual framework of the research.

As mentioned earlier and adapting from Meriläinen [8]'s model, the research conceptual framework is constructed as below (Figure 1). Based on the conceptual framework the research has eight hypotheses as follow:

- There are significant and negative relationship between high workload and academic burnout.
- There are significant and negative relationship between achievement motivation and academic burnout.
- There are significant and positive relationship between quality of teaching and learning environment and achievement motivation.
- There are significant and positive relationship between hope for employment and achievement motivation.
- There are significant and negative relationship between high workload and achievement motivation.
- There are significant and negative relationship between hope for employment and academic burnout.
- There are significant and negative relationship between academic quality of life and academic burnout.
- There are significant and positive relationship between academic quality of life and achievement motivation.

## 3 Method

### 3.1 Research design

This is a qualitative research in terms of paradigm and is a non-experimental research with an applied purpose. This is a correlational research that was done through a survey. It is a correlational research because measures and assesses the statistical relationship between different variables. In this regard and since structural equation modeling has been used to analyze the data a variance–covariance matrix was employed.

### 3.2 Participants

The statistical population of the study composed of the students of different educational levels in agricultural colleges of the state-run universities of Iran (N = 236,973). Accordingly, 386 students selected using Krejcie and Morgan [34]'s table and stratified random sampling with proportionate to size. We used a stratification system developed by the Ministry of Education for sampling. It should be noted that at the undergraduate level, since the junior and senior students can better answer the questions, sampling was done randomly among them. The statistics related to the exact number of students in each of the educational levels collected through correspondence with the education affairs of each university, and the sample size determined in proportion to the number of students at each level of education as presented in Table 1. The results of frequency distribution of respondents by gender showed that male students (204, 52.8%) constitute the majority of respondents. Female students comprise 182 (47.2%) of the sample size. Using independent sample t-test show that there is no difference between mean of academic burnout of male (M = 42.61) and female (M = 43.30) students (Sig = 0.25).

**Table 1:** Statistical population and sample size of research.

University	Statistical population				Sample Size			
	B.Sc.	M.Sc.	Ph.D.	Total	B.Sc.	M.Sc.	Ph.D.	Total
Tehran	2038	1226	595	3859	57	35	17	109
Razi, Kermanshah	1016	412	173	1601	29	11	5	45
Shiraz	2453	747	200	3400	69	21	6	96
Ramin, Ahwaz	1475	631	374	2480	42	18	11	71
Ferdowsi, Mashhad	1588	491	197	2276	45	14	6	65
Total	8570	3507	1539	13616	242	99	45	386

### 3.3 Measurement

The survey instrument is a questionnaire included 104 questions arranged in six sections related to measuring main variables. They were related to the assessment of academic burnout (14 questions) [24], workload (4 questions) [8, 29], academic quality of life (16 questions) [7], quality of teaching and learning environment (47 items) [8, 29], hope for employment (7 questions), and achievement motivation (16 questions) [8, 29]. Data was collected through a postal survey.

#### 3.3.1 The validity and reliability of the research instrument

A panel of agricultural faculty members established the face validity of the questionnaire. Average Variance Extracted (AVE), which used for discriminant validity, was also calculated. The components with AVE more than *0.50* [35] have acceptable validity. A pilot test was conducted for calculating the reliability of the questionnaire and Cronbach's alphas coefficients ranged from *0.75 to 0.84* and all were highly satisfactory. Due to the weaknesses of Cronbach's alpha method, including considering the same values for all statements of a component [40], the Composite Reliability (CR) was also calculated. The components with CR more than *0.70* [35] have acceptable reliability. The values of *α*, AVE and PC were presented in Table 2.

**Table 2:** Measurement model, Cronbach's alpha, factor loadings and discriminant.

Factor	Indicator	Definition	Items	α	AVE	PC
Quality of teaching and learning environmen	Teacher–student relationship	Quality of the environment which students attend. This environment includes the relationship between the teacher and the student, quality of teaching, teaching with the goal of deep learning, evaluation, educational counseling, expecting the success of the study, the social relationship between students, and applying of teaching in practice.	8	0.84	0.569	0.905
	Quality of teaching					
	Deep approach to learning					
	Evaluation					
	Pedagogical counselling					
	Psychological contract					
	Social relations between students					
	Usability of studies					
Achievement motivation	Ability beliefs	The wish or enthusiasm of a person to succeed and engage in activities which depends on the individual's efforts and ability, believing in one's own abilities, using lessons when he/she is employed, gaining his skills and knowledge during education that can use in the future and the expectation of success is defined by the study that he has done.	3	0.76	0.589	0.725
	Expectation of study success					
	Appreciation of university studies					
High workload	-	Students' activity in the course of study in the form of assignments and curriculum projects, etc.	-	0.75	0.578	0.721
Academic Quality of life	Science and Technology	Student satisfaction with educational technologies, classroom, academic popularity, popularity and reputation of professors, religious and spiritual programs of the university, sports space and recreational activities, library services, university transportation and parking services, self-service and food services.	4	0.81	0.565	0.837
	Accommodations					
	Extracurricular					
	Educational environment					
Hope for employment	Interest in the field of study	The amount of mental ability to meet a future job and develope ways to meet this goal, which includes two dimensions of interest in the field of study and social status of the field of study.	2	0.77	0.591	0.743
	Social status of the field of study					
Academic burnout	Exhaustion	The development of negative tendencies toward continuing education and increasing demands and work pressures for the student (emotional exhaustion), creating pessimistic attitudes and negative behaviors toward continuing education (anxiety and pessimism), and a low sense of competence and efficiency of the student in study (inefficiency).	3	0.76	0.563	0.792
	Cynicism					
	Inefficacy					

### 3.4 Data analysis

Data were analyzed using Structural Equation Modeling (SEM) technique and Maximum Likelihood Method. The Lisrel *8.50* software was used to estimate the model for research hypotheses. The Lisrel models comprise both measurement and structural models. In the measurement models the links between the latent variables and their indicators is investigated, whereas the structural models investigate the links between the latent variables. Relevant indicators (RMSEA, Chi-square, etc.) have been used to determine the goodness of fit of the model.

## 4 Results

### 4.1 Evaluation of the measurement model

In this section, we investigate the relationship between latent variables and their indicators. To determine the reliability and validity of the model, the level and significance level of the relationships between each latent variable with the relevant indicators was considered. In fact, the research model was reflective, in which the direction of causal relationship between the variable and the relevant indicators was from the variable to the indicators; and in terms of the correlation between the indicators of each variable, the indicators had a lot of correlation. In the reflective model of this study, it was expected that with the change in an indicator, the effects of the change will be reflected in all other indicators. The results of this analysis for the standardized value of the parameter, t value, standard error, and R^2^ are displayed in Table 3. Since parameters with t values greater than 1.96 are statistically significant [36], thus the results show that the indicators of the latent variables have an acceptable adaptation to the factor structure and theoretical basis of the research. The reliability of the indicators can be measured through the r-square of multiple correlations (R^2^).

**Table 3:** The measured standardized coefficients, significance level and confirmatory factor analysis of the latent variables.

Latent Variable	Indicators	lambda Parameter	Standard Error (SE)	t	R^2^
	Cynicism	0.81	0.068	11.89	0.65
Academic burnout	Inefficacy	0.80	0.067	11.86	0.65
	Exhaustion	0.63	-	-	0.39
Achievement motivation	Expectation of study success	0.87	0.071	12.09	0.76
	Ability beliefs	0.64	-	-	0.41
	Appreciation of university studies	0.52	0.062	8.73	0.27
High workload	The number of courses is high in proportion to my degree and the points I get.	0.76	0.050	14.59	0.57
	Sometimes major studies' workload is at times unbearable	0.66	0.051	12.82	0.44
	The hours that I go to the classroom are very uplifting.	0.57	0.053	10.67	0.32
	Workload in my subject is too much	0.053	0.53	9.89	0.28
Quality of teaching and learning environment	Quality of teaching	0.80	0.043	18.40	0.65
	Expectations from the teaching and learning environment	0.77	0.044	17.32	0.60
	Deep approach to learning	0.75	0.045	52.16	0.56
	Teacher–student relationship	0.70	0.046	15.19	0.49
	Evaluation	0.69	0.046	14.85	0.45
	Pedagogical counselling	0.64	0.047	13.35	0.41
Academic Quality of life	Accommodations	0.82	0.044	18.37	0.67
	Extracurricular	0.78	0.045	17.21	0.61
	Educational environment	0.71	0.047	15.05	0.50
	Science and technology	0.69	0.047	14.60	0.48
Hope for employment	Interest in the field of study	0.79	0.062	12.70	0.62
	Social status of the field of study	0.75	0.061	12.27	0.56

Based on Table 3, cynicism, volume of courses, accommodations, quality of teaching, interest in the field of study and expectation of study success have the most reliability for academic burnout, high workload, academic quality of life, quality of teaching and learning environment, hope for employment and achievement motivation, respectively. Therefore, the results show that the indicators had acceptable compliance with theoretical foundation of the research.

### 4.2 Fitting the structural model

In this section, the relationships between latent variables examined. The goal was to discover whether the theoretical relations between variables verified by the data. Accordingly, the research hypotheses were tested. To test the hypotheses, the path coefficient of variables on each other and t-value were calculated. The results were presented in Table 4. The results revealed that the hypothetical relationships between some latent variables were confirmed.

**Table 4:** Hypothesis**,** path coefficients and their significance values.

Path			Path coefficient	t- value	Sig.
High workload	→	Academic burnout	0.51	7.68	0.01
Achievement motivation	→	Academic burnout	-0.41	4.73-	0.01
Quality of teaching and learning environment	→	Achievement motivation	0.45	6.15	0.01
Hope for employment	→	Achievement motivation	0.35	5.88	0.01
High workload	→	Achievement motivation	-0.14	-2.62	0.01
Hope for employment	→	Academic burnout	0.08	1.21	Non-Significant
Academic quality of life	→	Academic burnout	-0.10	1.70-	Non-Significant
Academic quality of life	→	Achievement motivation	0.12	1.75	Non-Significant

**Figure 2:**
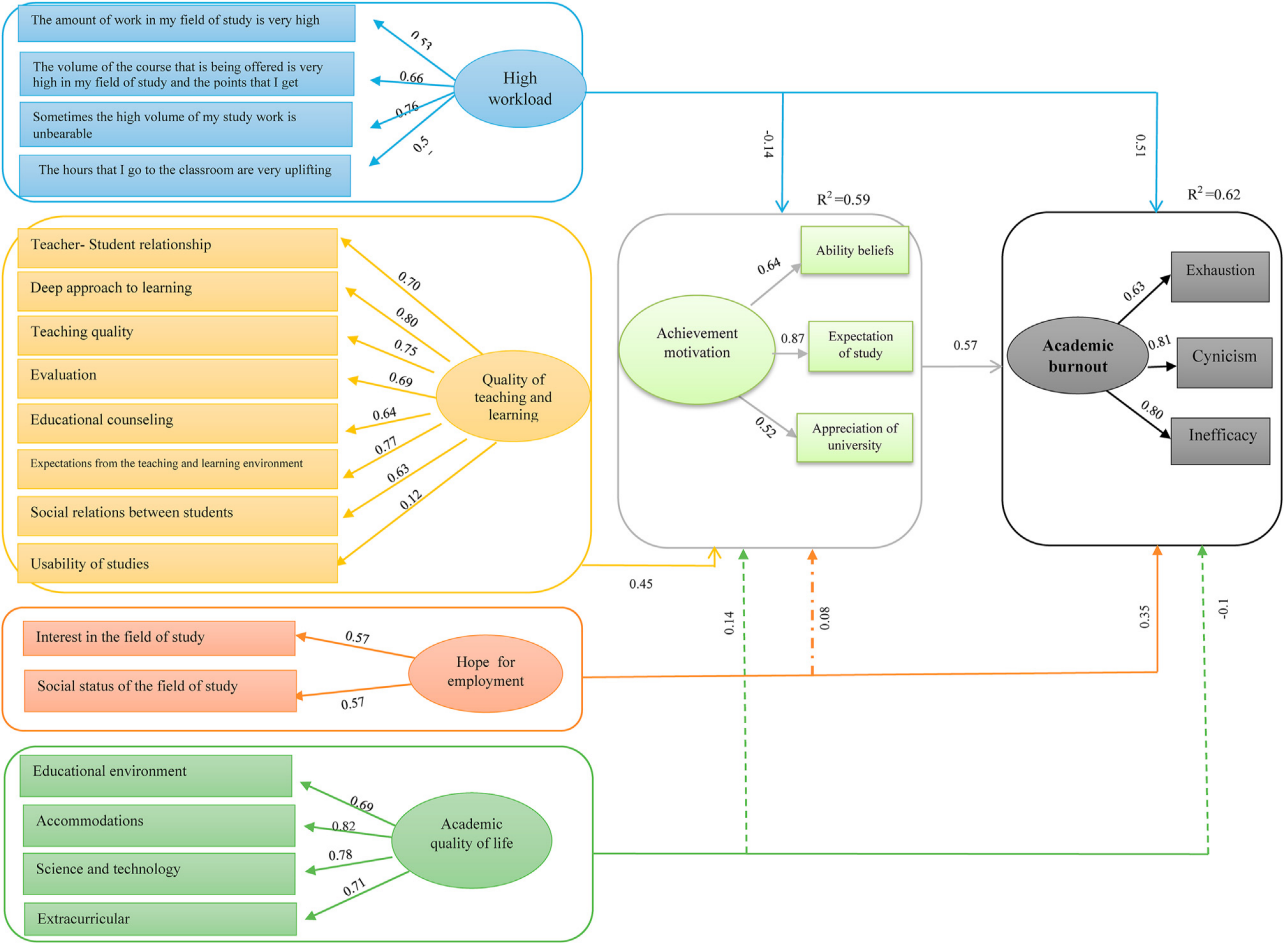
LISREL estimates of structural coefficients between variables and their indicators (empirical model).

The path coefficients presented in Table 4 confirm that among independent variables, high workload has the greatest effect on academic burnout. In addition, the values of R^2^ for the endogenous latent (dependent) variables of the model that indicated the effect of exogenous variables on the endogenous variables are also presented in Figure 2.

In fact, three values of 0.19, 0.33 and 0.67 were the criteria for weak, moderate and strong “R^2”^ [31]. Given the fact that the value of R^2^ was 0.59 for achievement motivation variable that is explained by high workload, quality of teaching and learning environment, hope for employment and academic quality of life. It was 0.62 for academic burnout variable that is explained by high workload, hope for employment, academic quality of life and achievement motivation. Thus, the appropriateness of fitting the structural model was confirmed. On the other hand, this means that 0.62 of the variations in variability of academic burnout are related to the four variables of high workload, hope for employment, academic quality of life, and achievement motivation.

According to path coefficient in Table 4, equation of the effect of high workload (HWL), hope for employment (HE), academic quality of life (AQL) and achievement motivation (AM) on academic burnout (AB), and the effect of proper workload (PWL), quality of teaching and learning environment (QTLE), hope for employment (HE), academic quality of life (AQL) on achievement motivation (AM) are as follow:

$$\begin{array}{ll} \text{AB}=0.51\;\text{HWL}-0.41\;\text{AM} & {\text{R}}^{2}=0.62\\ \text{AM}=-0.14\;\text{HWL}+0.45\;\text{AM}+0.35\;\text{HE} & {\text{R}}^{2}=0.59 \end{array}$$

### 4.3 Evaluation of the whole model

Finally, the goodness of fit statistics of the model were evaluated to find out how much the model is in agreement with data. For this purpose, goodness of fit indexes was calculated as in Table 5. As it is shown in Table 5, all the goodness of fit indexes has acceptable values. Therefore, the results show a reasonable fit between the model and the data. According to what was said, the empirical model of the research is shown below (Figure 2).

**Table 5:** Goodness of fit indexes.

Indexes	Acceptable amount	Reported amount
Chi square	-	2.873
Goodness of fit index (GFI)	0.90	0.98
Normed fit index (NFI)	0.90	0.94
Non-normed fit index (NNFI)	0.90	0.95
Incremental fit index (IFI)	0.90	0.92
Comparatives fit index (CFI)	0.90	0.96
Root mean square residual (RMR)	<0.08	0.038
Root mean square error of approximation (RMSEA)	<0.08	0.070

## 5 Discussion

In this research, an attempt was made to provide a comprehensive view of the components and their effect on students' academic burnout based on existing resources. To accomplish this research, eight hypotheses concerning the influence of the components on the increasing of students' academic burnout were formed and five of them were confirmed. Comparing different components, the results revealed that high workload had the most impact on increasing student's academic burnout. Students from different fields of agriculture stated that contrary to the working nature of their fields, a lot of theoretical assignments are given in these fields and this is a factor affecting their academic fatigue and their lack of interest in the field of agriculture. Given the high unemployment rate in agriculture, students are not interested in doing their homework because they find the field unfavorable and they show no interest to study in this field. The same results were found in earlier studies, e.g. Jacobs and Dodd [9]. But other researchers reported opposite findings [8, 28, 29]. Achievement motivation as second component had the most negative and direct effect on increasing student's academic burnout. Motivated students are less prone to burnout. These people participate in class activities with more interest, effort and perseverance and are confident in their ability to do homework. This result is in well agreement with other studies [8, 11, 28, 29, 30]. Motivated students do their homeworks volunteerily, try to do them better, and do not easily fall into academic burnout. Therefore, it is suggested that faculty members avoid academic burnout by encouraging students to engage in academic work and education that can create jobs for their future. Furthermore, evidences were found that three variables of quality of teaching and learning environment, hope for employment and academic quality of life had negative effects on increasing students' academic burnout through moderating role of achievement motivation.

Regarding the moderate variable, achievement motivation, the results of model fitting showed that high workload had a negative effect on the achievement motivation, so that the more the workload, the less motivated the student would be. Therefore, students should do different duties because they need enough facilities and resources. When the academic tasks are a lot and the students do not have enough resources, they are going to experience higher levels of stress, and their ability and motivation for doing these tasks will be declined. Therefore, teachers must reduce the amount of theoretical assignments that are irrelevant to their job prospects in order to motivate their students. They should give more practical assignments and entrepreneurial skills. In this regard, the curriculum content should be designed in such a way that attracts students' attention and be applicable in their daily lives. In addition, the assignments given to students should increase their skills related to the field and their future job. Therefore, the educational planners should revise the course syllabus and make the lessons more practical. The course syllabuses should also be run properly so that students learn their field skills. This finding is not agreement with the results of previous research [8, 28, 29].

Hope for employment had no direct effect on increasing academic burnout, which is not in line with previous research [26, 27]. On the other hand, it can be said that hope for employment had a direct effect on achievement motivation that is consistent with [26]. This states that the stronger the hope for the employment among students, the higher the motivation for achievement. Hope for employment leads to better performance in students 'educational activities and brings a positive attitude towards the field of study and students' interest in class activities and homework. According to the model, this variable affects academic burnout through the mediating variable of achievement motivation. The higher the hope for employment, the more interested students are in their field of study. Students who are uninterested and unmotivated in their studies are likely to not work hard enough, and their failure lead to academic burnout. In this regard, the agricultural higher education system should, in the first step, provide the circumstances for students to enter their favorite fields of study, and in the next stage, with the necessary planning, teach them the skills related to their field so that they will be able to find jobs related to their field of study. As mentioned the academic quality of life does not have a direct effect on academic burnout. This finding is not consistent with [12, 27]. Also, the results of the model fit showed that the academic quality of life has no direct effect on the achievement motivation. This finding is not consistent with [37]. Factors such as high workload, quality of teaching and learning environment, and hope for employment are more powerful than the academic quality of life. Despite these variables, the impact of academic quality of life is severely undermined. Contrary to the results of this study, the findings of other researchers have shown that students' quality of life plays an important role in their academic achievement and learning process. Students with appropriate quality of life can move away from academic burnout and achieve academic achievement by establishing stronger social networks, social support and emotional stability [4, 27].

Also, the results of model fitting show that quality of teaching and learning environment had a direct effect on achievement motivation. This result is in good agreement with other studies [8, 28, 29, 38].

Motivation can be effectively created by the relationship between teacher and student and teachers' paying attention to educational justice, introducing teachers to new teaching methods and improving the teachers' quality of teaching by holding in-service classes for them, providing appropriate feedback by the teachers to the students after evaluating and performing assignments. Also, applicability of the courses offered in the field of agriculture is another motivating factor due to the nature of the field. As undergraduate students in the last two years and MSc. and Ph.D. students need more communication to perform activities such as paper presentations, and doing thesis and dissertations, therefore, the availability of professors and adequate educational guidance and counseling will lead to the elimination of student stress.

## 6 Conclusion

The results of the research indicated a negative and significant relationship between high workload and achievement motivation and the student's academic burnout directly. Other variables such as hope for employment and academic quality of life also influence academic burnout through achievement motivation. Of course, it cannot be said that quality of academic life cannot affect academic burnout certainly. It is necessary to re-examine this issue in future studies. At the end, it is mentioned that studying the relationship between academic burnout and other related variables (e.g. stress, social support) is suggested to reach a wider attention. Then, educators should increase the selfefficacy level among their students to predict or control academic burnout. It can be gained by presenting some tasks which can enforce personal achievements. In addition, verbal reinforcement is the other method to reinforce selfefficacy. However, due to significant role of external factors in academic burnout (e.g. quality of learning experience), educational administartors are suggested to prepare enough equipment and resources such as equipped and updated libraries and computer sites. In general, the results of this study can be effective step to prevent academic burnout, as well as removing obstacles to dynamic academic achievement. For example, developing a regulation for reducing students' workload and directing their homework to practical activities related to the job market and society and closely monitoring its implementation is a simple solution for improving academic motivation and reduce student's academic burnout. Another suitable field for further research is the study of the effect of students' academic burnout on the performance of universities. It is noteworthy that managers in universities and educational institutions are not willing to change the educational system to increase the vitality and motivation of students; For this reason, research on the causes of unwillingness to change, especially the four pillars of education (teachers, students, curriculum and learning environment) in universities can also be a good ground for further research.

## Declarations

### Author contribution statement

M. T. Moghadam: Conceived and designed the experiments; Performed the experiments; Analyzed and interpreted the data; Contributed reagents, materials, analysis tools or data; Wrote the paper.

E. Abbasi: Conceived and designed the experiments; Analyzed and interpreted the data; Contributed reagents, materials tools or data; Wrote the paper.

Z. Khoshnodifar: Analyzed and interpreted the data; Wrote the paper.

### Funding statement

This work was supported by 10.13039/501100008257Tarbiat Modares University.

### Competing interest statement

The authors declare no conflict of interest.

### Additional information

No additional information is available for this paper.

## Footnotes

1 National examination to enter state-run universities of Iran.
